# With or without cells, that is the question!

**DOI:** 10.1002/jsp2.1010

**Published:** 2018-03-24

**Authors:** Mauro Alini, Robert Mauck, Daisuke Sakai

**Affiliations:** ^1^ AO Research Institute Davos Platz Switzerland; ^2^ McKay Orthopaedic Research Laboratory University of Pennsylvania School of Medicine Philadelphia Pennsylvania; ^3^ Department of Orthopaedic Surgery Tokai University School of Medicine Isehara Japan

It is with great enthusiasm and elation that we are inaugurating this first issue of *JOR Spine.* The rationale and reasons for creating this new journal are reported in our Editors' Introduction above. Here, we would like to share with you some research thoughts that we hope will contribute to bringing spinal research to the next quantum level during the coming decade. It is the intention of the editors, with these short editorials, to stimulate discussion and dialogue on relevant research topics, and to encourage submissions to *JOR Spine* that address these topics.

## 1 WITH OR WITHOUT CELLS, THAT IS THE QUESTION

Cells are pivotal during development and growth of the spinal motion segment. Much has been added to our understanding in the last decade concerning cellular phenotypes, origin, and cell types present in the intervertebral disc; however, several questions remain unanswered. For example, can mesenchymal stem cell differentiate into disc cells (and if so, which phenotypes can they achieve?) and what is the final disposition of the notochordal cells that initially form the spine, just to mention two. Certainly, with the newly available molecular tools, such as gene editing (CRISPR/Cas), it will be possible to obtain more knowledge about how specific genes modulate cell behavior, differentiation, and migration during development, growth and hopefully disc degeneration. An important associated question is: will this novel cellular information be relevant to disc regeneration approaches in humans, where cells are very limited in number and exist in a challenging environment? If the answer to this question is yes, then how might these cells be successfully deployed? The interrelated questions are: are cells necessary to regenerate/restore normal (within the appropriate age range) disc properties? And, if yes, by which mechanism?

These are fundamental scientific interrogations, which will have a tremendous impact on how our scientific community approaches human disc regeneration within the next years; with cells, such as mesenchymal stem cells (MSCs), progenitor cells, induced pluripotent stem cells (iPSCs), secretomes or without cells, using prosthesis, biomaterials, decellularized extracellular matrices, and three‐dimensional (3D) printing.

With cells, a major issue to clarify will be the exact role played by the cells injected or recruited into the disc tissues; will cells contribute directly to the regeneration or indirectly via their secreted chemokines? If cells are actually needed to promote regeneration, then the associated processes, such as cellular differentiation, growth factors, and extracellular matrix synthesis will need to be investigated within a hostile environment (there are not many reports on the fate of cells placed within a degenerated disc; will they survive?); To the contrary, if regeneration is based on what cells are secreting then, theoretically secretomes could be used therapeutically. But, here again, the task of preparing such chemokines cocktails is not trivial, as this requires exposing “the productive cells” to the real human hostile disc environment to collect the appropriate secretome.

For approaches that do not rely on cells, we are presently not any better positioned. Artificial discs have failed, as have injectable hydrogels. The ideal glue for annulus repair is still a holy‐grail and there is nothing new on the biomaterial horizon! Is additive manufacturing a potential alternative? In theory yes, as you can print anything (especially without cells) in any shape; and a potential source of printable material could be the decellularized matrix of the disc. However, how does one formulate decellularized matrix into a printable format, without losing the intrinsic biochemical and biomechanical properties? And how you will surgically exchange the patient's degenerated disc with the printable disc?

The final answer may not be with or without cells, given that different disc afflictions will need alternative treatments. For sure, by better understanding the pathological process (or processes) of disc degeneration (particularly in humans) new, presently unknown, regenerative opportunities will emerge, hopefully leading to improved outcomes, which is extremely desirable to augment our currently limited clinical armamentarium against disc degeneration.

